# Amelioration of experimental autoimmune encephalomyelitis by in vivo reprogramming of macrophages using pro-resolving factors

**DOI:** 10.1186/s12974-023-02994-5

**Published:** 2023-12-20

**Authors:** Thierry Gauthier, Omayra Martin-Rodriguez, Cécile Chagué, Anna Daoui, Adam Ceroi, Alexis Varin, Francis Bonnefoy, Séverine Valmary-Degano, Mélanie Couturier, Susanne Behlke, Philippe Saas, Pierre-François Cartron, Sylvain Perruche

**Affiliations:** 1 Université de Franche-Comté, EFS, INSERM, UMR RIGHT, 25000 Besançon, France; 2 Pathology Department, Besancon University Hospital, 25000 Besançon, France; 3 Team “Apoptosis and Tumor Progression” CRCINA-INSERM U1232, Université de Nantes Nantes, LaBEX IGO, REpiCGO, EpiSAVMEN, LaBCT, Institut de Cancérologie de L’Ouest (ICO), 44000 Nantes, France; 4 MED’INN’Pharma, 25000 Besancon, France

**Keywords:** Resolution of inflammation, Neuroinflammation, Macrophages, Epigenetic reprogramming, Secretome

## Abstract

**Background:**

Reinstating inflammation resolution represents an innovative concept to regain inflammation control in diseases marked by chronic inflammation. While most therapeutics target inflammatory molecules and inflammatory effector cells and mediators, targeting macrophages to initiate inflammation resolution to control neuroinflammation has not yet been attempted. Resolution-phase macrophages are critical in the resolution process to regain tissue homeostasis, and are programmed through the presence and elimination of apoptotic leukocytes. Hence, inducing resolution-phase macrophages might represent an innovative therapeutic approach to control and terminate dysregulated neuroinflammation.

**Methods:**

Here, we investigated if the factors released by in vitro induced resolution-phase macrophages (their secretome) are able to therapeutically reprogram macrophages to control neuroinflammation in the model of experimental autoimmune encephalomyelitis (EAE).

**Results:**

We found that injection of the pro-resolutive secretome reduced demyelination and decreased inflammatory cell infiltration in the CNS, notably through the in vivo reprogramming of macrophages at the epigenetic level. Adoptive transfer experiments with in vivo or in vitro reprogrammed macrophages using such pro-resolutive secretome confirmed the stability and transferability of this acquired therapeutic activity.

**Conclusions:**

Overall, our data confirm the therapeutic activity of a pro-resolution secretome in the treatment of ongoing CNS inflammation, via the epigenetic reprogramming of macrophages and open with that a new therapeutic avenue for diseases marked by neuroinflammation.

**Supplementary Information:**

The online version contains supplementary material available at 10.1186/s12974-023-02994-5.

## Background

Autoimmune disorders are often of complex and unknown etiology but are marked by alterations of the resolution phase of inflammation, leading to uncontrolled or non-resolving chronic inflammation responsible for continuous tissue damage. Multiple sclerosis (MS) is a demyelinating chronic inflammatory disease of the central nervous system (CNS) in which pro-inflammatory leukocytes infiltrate the CNS, leading to the destruction of the myelin sheath and eventually to the loss of neurons [1]. It has been recently shown that innate immune cells are crucial for the development of this pathology and that these cells are a key component of the inflammatory response [1]. More importantly, innate immune cells (specifically macrophages) control the resolution of inflammation and the return to homeostasis, a process which is altered during the development of multiple sclerosis. There is reported evidence of the accumulation of neutrophils in the periphery related to enhanced CXCL1/IL-8 production in experimental autoimmune encephalomyelitis (EAE) and in MS patients [2]. Furthermore, macrophages are the most abundant blood-derived cell types in acute inflammatory MS lesions[1] and selected specialized pro-resolving lipid mediators (SPMs), involved in resolution phase [3], are reduced in the cerebrospinal fluid and peripheral blood of MS patients [4–6]. Additionally, the expression of the pro-resolving mediator, developmental endothelial locus-1 (DEL-1) [7] is also decreased in chronic-active MS lesions and during the course of EAE [8]. A deficiency and/or dysfunction of regulatory T cells (Treg) is observed during this disease [9], while the suppressive activity of IL-10-secreting regulatory T 1 (Tr1) cells is impaired in MS patients [10]. Some innovative approaches fostering resolution of inflammation have been recently developed [11–13]. These are based on the administration of SPMs [14], the induction of T cell apoptosis in vivo using anti-CD3 monoclonal antibody infusion [15], apoptotic cell infusion in association with pathogenic auto-antigen [16], or induction of cell apoptosis in vivo in the presence of the pathogenic auto-antigen [17]. However, these approaches demonstrate short-term tolerance induction. In some of these therapeutic developments [16, 17], the pathogenic auto-antigens that trigger MS needs to be identified, potentially limiting their transfer into the clinic.

Resolution of inflammation promotes the return to homeostasis, with tissue healing and repair [18]. Many molecules are implicated in this process. Notably, SPMs are associated with the shift from inflammation to resolution [3], as well as anti-inflammatory cytokines which participate in macrophages reprogramming [19, 20]. Other molecules can directly inhibit or degrade pro-inflammatory lipids or cytokines in order to limit further inflammatory cell recruitment and over-inflammation [21]. Most of these molecules are produced after efferocytosis, i.e., the elimination of apoptotic cells by phagocytes, and in particular by tissue-resident and monocyte-derived macrophages [22, 23]. While efferocytosis of dying cells per se contributes to tissue cleaning, restoration of homeostasis and initiation of tissue repair, the molecules released by macrophages during this process enhance inflammatory response deceleration and arrest, [22–25]. This suggests that molecules released during efferocytosis, and captured as a pro-resolutive secretome, might represent an innovative therapeutic approach to restore or reinitiate natural resolution of inflammation to terminate chronic inflammation [11, 13, 18]. The secretome of pro-resolving factors released by macrophages after efferocytosis contains a large number of molecules including chemokines (CCL5, CXCL2, and CCL22), anti-inflammatory cytokines (IL-1RA, IL-10, and TGF-β) [26], growth factors (TGF-β, IGF-I and VEGF) [27], enzymes, metabolites and polyunsaturated fatty acids, that have been demonstrated to endow a therapeutic activity to control experimental collagen-induced arthritis [26], dextran sodium sulfate (DSS)-induced and T cell transfer-induced colitis [27]. In these settings, pro-resolving factors induced autoantigen-specific Treg via the reprogramming of antigen-presenting cells (APC) including macrophages [26] and enhanced tissue repair [27].

In this study, we aimed to investigate if the use of pro-resolving factors also promotes macrophage reprogramming therefore impacting CNS inflammation and its related disease activity. We demonstrated that the intraperitoneal (i.p.) injection of pro-resolving factors resolved ongoing inflammation in myelin oligodendrocyte glycoprotein peptide 35–55 (MOG_35-55_)-induced EAE. This beneficial effect was mediated by in vivo reprogramming of macrophages toward a pro-tolerogenic/pro-resolving profile. In vivo depletion of macrophages using clodronate-loaded liposome injection before treatment abolished the resolution of EAE. In contrast, the adoptive transfer of reprogrammed macrophages isolated from treated EAE mice or generated in vitro, limited EAE progression in EAE mice, induced Treg increase in the spinal cord and a reduction of pathogenic Th1 cells in the inguinal draining lymph nodes (iLN). Importantly, CD11b^+^ myeloid cells, notably macrophage reprogramming occurred through deep epigenetic modifications increasing the expression of the anti-inflammatory *miR-223*. Thus, the administration of pro-resolving factors represents a new therapeutic approach to restore inflammation resolution to manage chronic inflammatory diseases.

## Materials and methods

### Mouse experimentation

Female C57BL/6J mice aged of 6–10 weeks were purchased from Charles River Laboratories. OTII/RAG^–/–^ mice, kindly provided by Dr Helena Paidassi, were bred in our animal facility. Experimentations (#02831) were approved by the local Animal Ethics Committee of Besançon (*Comité d’Ethique Bisontin en Experimentation Animale* #58), the French Ministry of Higher Education, Research and Innovation (*Ministère de l’Enseignement Supérieur, de la Recherche et de l’Innovation*) and the French Ministry of Agriculture (experimentation protocols are registered in APAFIS system under the number 2021-004-OA-12PR). These experiments were conducted in accordance with the European Union’s Directive 2010/63. Mice were housed at the environmentally controlled and pathogen-free UMR1098 animal facility (#D25-056-7) in ventilated cages with cellulose bedding and access ad libitum to pellet food and sterile water pouches (Plexx, The Netherlands).

### Production of SuperMApo

Briefly, as previously described [26–28], C57BL/6J mice peritoneal macrophages were cultured with apoptotic thymocytes and the culture supernatant was collected at 48 h and stored at − 80 °C until use. Supernatant of macrophages or apoptotic cells cultured alone were also generated as controls.

### EAE induction and SuperMApo treatment

C57BL/6J female mice were injected subcutaneously with 200 µg of MOG_35-55_ peptide (MD Bioproducts) emulsified in IFA with 5 mg/mL of heat-inactivated *Mycobacterium tuberculosis* (Sigma-Aldrich). Pertussis toxin (Calbiochem) was injected i.p. at 0 and 48 h after disease induction (200 ng/mouse). Mice were monitored and scored daily as follows: 0: no disease; 1: flaccid tail; 2: hind limb weakness; 3: partial hind limb paralysis; 4: total hind limb paralysis; 5: moribund state or death. When mice reached a 0.5–1 clinical score, SuperMApo or vehicle (X-vivo medium) were injected i.p. (1 mL) and reinjected 48 h later.

### Cell isolation and staining

Spinal cords were harvested without prior perfusion, washed in PBS and incubated with type II collagenase (2 mg/ml; Roche). Then, mononuclear cells were separated using a 30% Percoll layered over a 70% Percoll (Sigma-Aldrich) solution by centrifugation (600*g*, 20 min). Spleens and inguinal lymph nodes were harvested and cells extracted and stained with a cell viability dye FvS (BD biosciences), as well as labeled monoclonal antibodies against CD45 (BD biosciences, clone 30-F11), CD11b (BD biosciences, clone M1-70), CD11c (BD biosciences, clone HL3), CD19 (BD biosciences, clone 1D3) or CXCR3 (BD biosciences, clone CXCR3-173), F4/80 (BD biosciences, clone T45-2342) after pre-incubation with anti-Fc receptor antibody (clone 24G2). Expression of co-stimulation markers was assessed using CD80 (BD biosciences, clone 16-10A1), CD86 (Biolegend, clone GL-1), CD40 (Biolegend, clone 3/23) and MHC-II (Miltenyi Biotec, clone REA813) antibodies. For intracellular staining, cells were stimulated with 1 µg/mL of LPS (or not) (Sigma-Aldrich, only for in vitro stimulation assays) for 6 h and then with 25 ng/mL of phorbol 12-myristate 13-acetate (Sigma-Aldrich) and 1 µg/mL of ionomycin (Sigma-Aldrich) in presence of 10 µg/mL of monensin (BD biosciences) for 4 h at 37 °C. Foxp3 (ebiosciences, clone FJK-16S) expression as well as intracellular TNF-α (BD biosciences, clone MP6-XT22), IL-12 (BD biosciences, clone C15.6), IL-10 (BD biosciences, clone JES5-16E3), IL-17A (Miltenyi Biotec, clone REA660), IFN-γ (Miltenyi Biotec, clone REA381) or KI-67 (BD biosciences, Clone B56) staining were performed in CD3^+^ (BD biosciences, clone 145-2C11), CD4^+^ (BD biosciences, clone RM4-5), CD8^+^ (BD biosciences, clone 53-6.7)-gated cells, using a FACS CANTO II cytometer with DIVA v7 software (BD biosciences).

### T cell recall responses

Inguinal lymph nodes and spleen cells were harvested and stimulated with 15 µg of MOG_35-55_ peptide for 72 h. Cell proliferation was evaluated by BrdU incorporation and counting following manufacturer instructions (Perkin Elmer) and T cell polarization was evaluated by FACS.

### Suppressive assays

For MOG-specific or *Mycobacterium tuberculosis* (MT)-specific co-cultures, naive CD4^+^CD25^‒^ T cells were isolated from EAE mice by MACS (Miltenyi Biotec) and cultured (100.10e3 cells) with CD11c^+^ dendritic cells (DC) (50.10e3 cells) isolated from naive mice (CD11c MicroBeads, Miltenyi Biotec), in the presence of 15 μg/mL of MOG_35-55_ peptide (MD Bioproducts) or 50 µg/mL MT protein (Difco). For CD3/CD28-stimulated T cell cultures, CD25^–^CD4^+^ naive T cells were isolated from EAE mice and cultured in the presence of coated anti-CD3 (BD biosciences, clone 145-2C11) and soluble anti-CD28 (BD biosciences, clone 37.51) antibodies (2 and 0.5 μg/mL, respectively). After adding sorted Treg and after 4 days of culture, T cell proliferation was assessed by BrdU incorporation and counting. Cell proliferation is depicted as a ratio of the BrdU incorporation level of MOG-stimulated cells divided by the BrdU incorporation level of non-stimulated cells (medium). Ratios were then normalized to the control group (EAE).

### RNA isolation, cDNA reverse transcription and RT-qPCR

Total RNA from CNS, spleen and iLN cells were isolated using the RNA easy mini kit (Qiagen) according to manufacturer’s protocol and used for first-strand cDNA synthesis (Applied Biosystems). Then, mRNA expression levels were quantified by real-time quantitative PCR using Fast SYBR Green Master Mix (Applied Biosystems) with CFX96 system (Biorad). The primers (ThermoFisher) used for the assay are: *Ccl2* (Mm00441242), *Ccl5* (Mm01302427), *Claudin-5* (Mm01169675_s1), *Cxcl9* (Mm00434946), *Cxcl10* (Mm00445235), *Cxcr3* (Mm99999114), *Ccr5* (Mm01963251), *Ccr6* (Mm99999054), *F11r* (Mm00554113_m1), Gapdh, *Icam* (Mm00516023_m1), *Itga4* (Mm01277951_m1), *Itgb5* (Mm00439825_m1), *Itgb7* (Mm00442916_m1), *Occludin* (Mm00500912_m1), *Tjp1* (Mm00493699_m1) and *Vcam1* (Mm01320970_m1).

### Histology

Spinal cords were extracted at killing, without perfusion, washed in PBS and fixed in 5% formol solution before being embedded in paraffin and sectioned. Sections (2 µm) were stained with Luxol fast blue or H&E (Merck) using standard procedures. Inflammation was evaluated by a pathologist blinded to the nature of the mice, and determined using a score system with three levels (0 = no infiltrate, 1 = low mononuclear cell infiltrate, 2 = middle mononuclear cell infiltrate, 3 = dense mononuclear cell infiltrate penetrating in the tissue). Demyelination was also evaluated and determined using a score system with three levels (0 = no demyelination, 1 = few areas of demyelination, 2 = middle areas of demyelination, 3 = large areas of demyelination).

### Blood brain barrier permeability evaluation

Evans Blue dye (Sigma-Aldrich) was injected i.v. at 72 h post SuperMApo or control treatment for 60 min and then mice were killed, the spinal cords removed and weighted. Evans Blue was extracted with 2.5 mL of 60% trichloroacetic acid, centrifuged for 30 min at 10,000*g* and the amount of Evans Blue was quantified at 610 nm by spectrophotometry and normalized to the spinal cord weight.

### Macrophage analysis

CD11b^+^ macrophages were sorted from the spleen using CD11b microbeads and an autoMACS pro device (Miltenyi Biotec) (purity > 90%). Cells were conserved in RLT buffer for RNA extraction, or cultured with naive CD25^–^CD4^+^ T cells issued from RAG^–/–^OT-II mice (50,000 APC for 100,000 T cells) in presence of OVA_323-339_ peptide (2 µg/mL; Invivogen). After 72 h of culture, T cell polarization was analyzed by FACS. In some experiments, homemade clodronate-loaded or PBS-loaded liposomes [29] were injected i.v. 24 h before SuperMApo treatment (2 mg per mouse). For adoptive transfer of macrophages, spleen CD11b^+^ cells were sorted using CD11b microbeads as specified above and injected immediately in EAE mice (5.10e6 cells per mouse) exhibiting a clinical score, as indicated. In other experiments, spleen CD11b^+^ cells were sorted from naive mice, treated ex vivo with vehicle (X-vivo medium) or SuperMApo for 24 h and injected in EAE mice (1.5.10e6 cells per mouse).

### Injection of CFSE-stained cells

Spleen from vehicle- or SuperMApo-treated EAE mice were removed 72 h after treatment and stained with CFSE (Thermo Fisher Scientific) for 10 min at 37 °C, washed and injected i.v in EAE mice (50 million per mice). Four h after the injection, percentage of CFSE^+^ CD11c^+^CD11b^‒^ DC, CD19^+^CD3^‒^ B cells and CD3^+^CD19^‒^ T cells in the spinal cord, iLN and spleen was assessed by FACS.

### Immunofluorescence

Spleen macrophages were sorted and stimulated with LPS for 1 h. Cells were then fixed in 4% paraformaldehyde (Sigma-Aldrich) and permeabilized with 0.1% triton X-100 (Sigma-Aldrich) at room temperature. Immunofluorescence staining was performed, as previously described [30, 31]. Briefly, non-specific sites were blocked with 0.1% Tween-TBS with 5% BSA for 1 h at 37 °C. Incubations with primary antibodies were performed overnight at + 4 °C, and then cells were rinsed 3 times with 0.1% Tween-TBS. Incubations with secondary antibodies were performed for 1 h at 37 °C and then cells were rinsed 3 times with 0.1% Tween-TBS, stained with 4',6'-diamidino-2-phénylindole (DAPI) and mounted using Vectashield mounting medium (Vector Laboratories). Primary antibodies were directed against pP65 (cell signaling; 3033S), P65 (cell signaling; 6956P), pIKBα (cell signaling; 9246 s), pIKKβ (abcam; ab192440) and secondary antibodies were directed against donkey anti-mouse IgG Alexa Fluor 555-conjugated (Life technologies; A31571) and donkey anti-rabbit IgG NorthernLights493-conjugated (R&D systems; NL006) antibodies. Mean fluorescence intensity (MFI) was determined using image J software as previously described [32].

### Transcriptomic analysis

RNA samples were sent to Integragen for sequencing on the NGS platform (Illumina HiSeq 4000) and the resulting raw data (fastq) were sent to the CLIPP platform (Dijon, France) for analysis on their custom pipeline (mouse reference genome alignment was performed using TopHat2 algorithm followed by differential expression of transcripts with Cufflinks algorithms). Resulting RNA-seq data, containing FPKM (Fragments Per Kilobase of exon per million fragments Mapped) values, were log_2_ transformed, normalized and mean-centered using Cluster 3.0 software and data visualization was performed using GiTools and TreeView 3 for the different Heatmaps, and Cytoscape 3.2 for network analysis of the associated pathways.

### Epigenetic studies

DNA extraction was performed using QIAamp DNA Mini QIAcube Kit and QIAcube (Qiagen, France). 5-methylcytosine ELISA was performed using 5mC ELISA (Ozyme/Zymo, France). TET activity was determined using eth 5mC-Hydroxylase TET Activity/Inhibition Assay Kit (Euromedex, France). DNMTs magnetic bead (DMB) assays were performed as described by Yokochi and Robertson (PMID: 15273420). A typical methylation reaction contained 30 nM of the respective DNMT protein (Methylation Ltd, Port Orange, Florida or Tebu-Bio, France), 125 nM DNA oligonucleotides, and 900 nM tritium-labeled AdoMet (Amersham Bioscience, 1 mCi/mL), in reaction buffer (50 mM Tris, pH 8.0, 5 mM EDTA, 10% glycerol, 0.5 mM phenylmethylsulfonyl fluoride). After incubation at 37 °C for 1 h, the reaction was quenched with an equal volume of magnetic beads suspension and incubated for 15 min at room temperature. Next, the beads were magnetically isolated from the reaction mix, and tritium incorporation was measured by scintillation counting. Unmethylated double-stranded oligonucleotides were used to estimate the de novo MTase activity, and hemimethylated double-stranded oligonucleotides were used to estimate the maintenance MTase activity.

miRNA extraction was performed using miRNeasy Mini Kit and QIAcube (Qiagen, France). miScript II RT Kit (Qiagen, France), miScript SYBR Green PCR Kit (Qiagen, France) and miScript Primer Assay were used to quantify the miRNA expression. MSRE methodology was adapted from the OneStep qMethylTM Kit (Ozyme, ZYMO, France). A typical digestion of genomic DNA contained 20 ng of DNA and 5 units of considered enzyme (New England Biolab, France). For the non-enzyme control, distilled water was added instead of *considered enzyme*. Except for certain enzymes, all prepared samples were incubated at 37 ºC for 12 h, followed by heat inactivation at 65 ºC for 20 min. qPCR was performed using SYBR Green PCR Kit (Qiagen, France). The methylation level for any amplified region was determined using the following equation %methylation = 100 × 2^−ΔCt^. Primers used are: miR127#1: GCGATGGAACTGAGTTTTGTC and TCCTGGTCTACTCAATGAG, miR127#2: GCTCAGAGGGCTCTGATTCAGAAA and ATGAGACTTCCGACCAGCCAA, miR-223#1: ACCAGGGTAAACAGAGCATACAAG and GTGGCCCAAGCCTCAATTAACTCT, and miR-223#2: ACACGAAACGTAACACTACA and CCAACTCAACTTATCAAATACACG.

Chromatin ImmunoPrecipitation (ChIP) experiments were performed with the LowCell#ChIP kit (Diagenode, France). Bioruptor (Diagenode, France) was used for the sonication step (3 runs of 8 cycles (30 s “ON”, 30 s “OFF”) at high power setting). Six μg of antibody (Anti-CTCF antibody (ab70303) Abcam, France) were used and IP tubes were incubated at 40 rpm on a rotating wheel for 4 h at 4 °C. Quantitative PCR were performed using the SYBR Green PCR Kit (Qiagen, France). Typically, TET conversion activity was determined by measuring the 5mC level (ELISA) in a methylated oligonucleotide probe mimicking a DNA region of interest. Biotinylated probe was incubated with 10 ng of Nuclear Extraction Kit (ab113474, Abcam, France) for 90 min at 37 °C in presence or not of 10 μg of considered antibody (cEBPα: sc- sc-9314, Santa Cruz, France, EBF1 (clone 1G8, Abnova, France), and FOXA1 Abcam ab24738). After isolation and washes, reagents issued from the MethylFlashTM Global DNA Methylation (5-mC) ELISA Easy Kit (Colorimetric) were used to detect the 5mC on probe.

### Statistical analysis

All data were analyzed for statistical significance using GraphPad Prism version 7.04 (GraphPad Software, San Diego, CA, USA) by adapted Student’s t test or one or two-way ANOVA test including multiple comparison post-tests, as indicated.

## Results

### Administration of pro-resolving factors modulate ongoing EAE through reduction of pathogenic T cells and macrophages infiltration

**Fig. 1:**
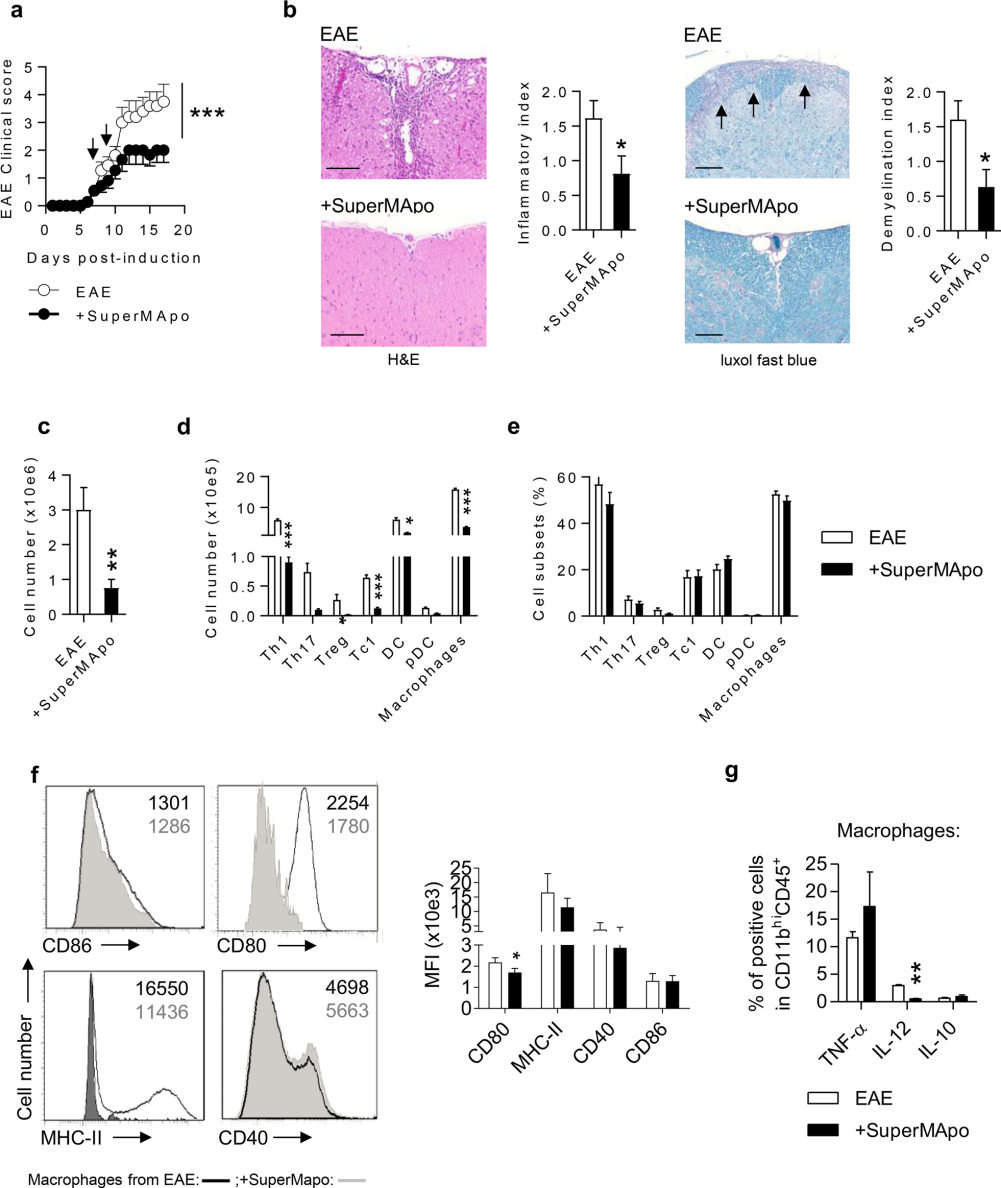
SuperMApo treatment attenuates ongoing MOG-induced EAE in C57BL/6 mice. **a** Clinical score of EAE mice treated twice (black arrows) with SuperMApo (*n* = 6 mice) or with vehicle (*n* = 5 mice). Data from a representative experiment out of 3 showing similar results. **b** Representative H&E and Luxol Fast Blue stainings of spinal cords 72 h after the first injection of SuperMApo or vehicle. Spinal cord inflammation in mice is evaluated by inflammatory index and scored in H&E sections. Demyelination is scored in Luxol Fast Blue sections. Demyelinated areas are identified with black arrows. Scale bar, 80 µM. Data pooled from 3 independent experiments (*n* = 15 mice per condition). **c** Total number of CD45^+^ leukocytes in spinal cords 72 h after the first SuperMApo (n = 5 mice) or vehicle (*n* = 5 mice) treatment. Data from a representative experiment out of 3 showing similar results. **d** Total number of Th1 (IFN-γ^+^CD4^+^CD3^+^), Th17 (IL-17^+^ CD4^+^CD3^+^), Treg (Foxp3^+^CD25^+^CD4^+^CD3^+^), Tc1 (IFN-γ^+^ CD8^+^CD3^+^), dendritic cells (DC) (CD11c^+^CD11b^−^), plasmacytoid DC (mPDCA^+^CD11c^+^CD11b^−^) and macrophages (CD11b^hi^CD45^+^). Data from a representative experiment out of 3 showing similar results. **e** Percentages of cells depicted in **d**. **f** Ex vivo analysis of macrophages co-stimulation and class-II molecule expression issued from SuperMApo or vehicle-treated EAE mice as MFI (**f**), as well as inflammatory cytokine content (**g**). Data pooled from 4 independent experiments, 20 mice per condition. Statistical significance was assessed by using ANOVA two-way and Bonferroni posttest (**a)** or unpaired two-tailed Student’s *t* test (**b**–**g**) (*p < 0.05; **p < 0.01; ***p < 0.001)

SuperMApo was injected i.p., fractioned in two doses at a 48 h interval, in mice with ongoing EAE exhibiting a clinical score of 0.5–1 out of 5. The days following SuperMApo treatment, we observed the immediate arrest of EAE progression, compared to the control group receiving vehicle, whose clinical score continued to increase (Fig. 1a). Consistent with this, SuperMApo-treated mice demonstrated a lower inflammatory score within the spinal cord, with reduced inflammatory cell infiltrates and lower demyelination 72 h post-treatment (Fig. 1b). The therapeutic control of ongoing EAE was only achieved using SuperMApo, but not when pooled factors from separate cultures of apoptotic cells and macrophages were used (Additional file 1: Fig. S1).

To better understand the treatment impact on the immune environment, we analyzed leukocyte subsets in the spinal cord extracts (not perfused) three days post-treatment. Consistently with immunohistochemistry analysis (Fig. 1b), we found that SuperMApo injection significantly decreased the total number of leucocytes in the CNS (Fig. 1c). This included Th1, Th17 and Tc1 cell subsets, dendritic cells (DC) and macrophage numbers (Fig. 1d), the latter being identified as CD45^+^CD11b^high^ cells (Additional file 1: Fig. S2a, b). However, the number of CD11b^med^CD45^+^ microglial cells, also known as CNS-resident macrophages, was not impacted (Additional file 1: Figs. S2a, b and S3a). Hence, SuperMApo treatment reduced inflammatory cell numbers but did not modify the representation of each subset (Additional file 1: Figs. S1e and S2a for gating strategy) in diseased tissues. While CNS quantities of inflammatory cytokines TNF-α, IL-12p70, IFN-γ, IL-6 and IL-10 were not modified by the treatment (Additional file 1: Fig. S3c), macrophages expressed significantly lower levels of CD80 and MHC class-II molecules, as well as reduced intracellular IL-12 content after SuperMApo treatment (Fig. 1f, g). Compared to macrophages, microglial cells did not demonstrate any differences in terms of co-stimulatory and MHC class-II molecule expression or cytokine content in the CNS after SuperMApo treatment (Additional file 1: Fig. S3d, e).

These data demonstrate that the secretome of resolution-type macrophages is able to stop the disease progression during ongoing EAE and decrease tissue inflammation through decreased pathogenic T cell infiltration and reduced CNS macrophage infiltration and activation.

Because SuperMApo treatment diminished the overall pathogenic cell infiltration in the spinal cord, we investigated the permeability of the brain blood barrier (BBB), which is known to be damaged by inflammation [33, 34]. Using intravenous Evans Blue (EB) dye injection (only passing the BBB when compromised) at 72 h post-treatment, we observed the same quantities of EB dye in the CNS suggesting that the BBB permeability was not impacted by SuperMApo treatment (Additional file 1: Fig. S4a). This was further supported by the quantification of mRNA levels of the major genes coding for proteins implicated in the integrity of the BBB, i.e., occludin, claudin-5, tight junction protein 1 and F11 receptor (*ocln*, *cldn5*, *tjp1* and *f11r*, respectively). SuperMApo treatment did not modulate the mRNA levels of these proteins (Additional file 1: Fig. S4b). In addition, no modification of chemokines and integrins expression at the CNS level was observed to explain the reduced CNS cell infiltrate after SuperMApo treatment (Additional file 1: Fig. S4c,d). Overall, our results show that the decreased CNS inflammation induced by SuperMApo treatment is not associated with BBB remodeling, but might be associated with the modulation of peripheral inflammatory immune cells.

### Administration of pro-resolving factors decreases the peripheral pathogenic T cell response

**Fig. 2:**
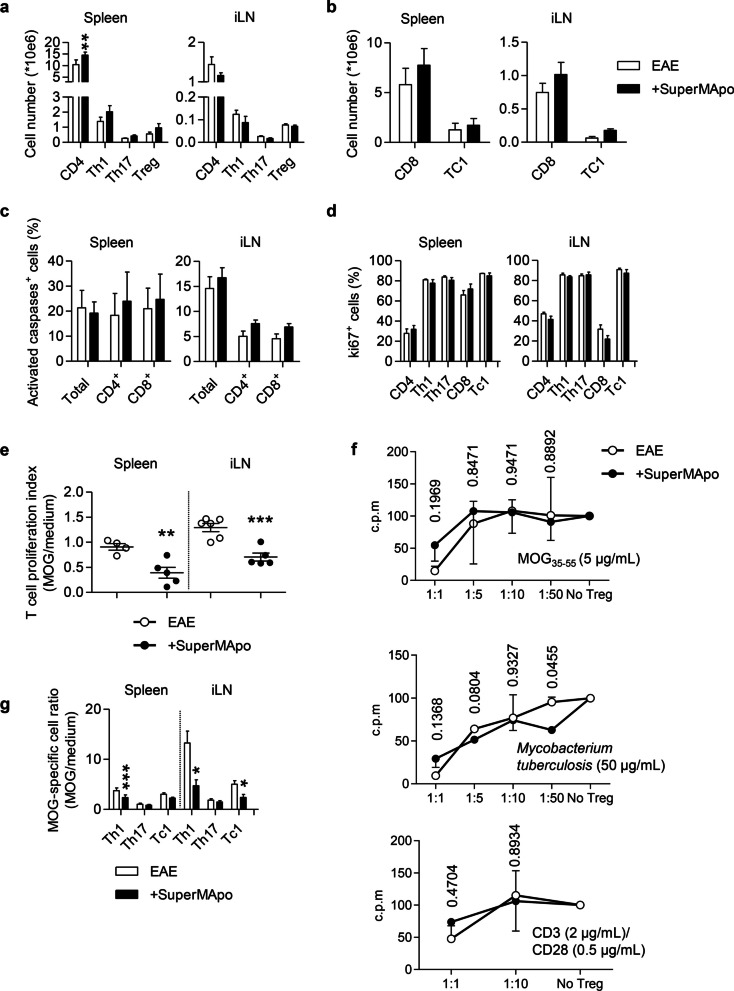
SuperMApo treatment decreases peripheral pathogenic T cell response. Absolute numbers of CD4, Th1, Th17, Treg (**a)** or CD8 and Tc1 (**b)** in spleen and inguinal lymph nodes (iLN) 72 h after the first SuperMApo treatment (+ SuperMApo) or vehicle (EAE) (*n* = 5 mice per group). Data from a representative experiment out of 3 showing similar results, shown as mean ± SEM. **c** Percentages of activated caspase-positive cells in CD4^+^CD3^+^, CD8^+^CD3^+^ or total leukocytes in the spleen (*n* = 3 mice per group). Data from a representative experiment out of 2 showing similar results. **d** Percentages of ki67-positive cells in CD4^+^CD3^+^, CD8^+^CD3^+^, IL17^+^CD4^+^CD3^+^ (Th17), IFN-γ^+^CD4^+^CD3^+^ (Th1) and IFN-γ^+^CD8^+^CD3^+^ (Tc1) in the spleen and iLN (*n* = 5 mice per group). Data from a representative experiment out of 2 showing similar results shown as mean ± SEM. **e** SuperMApo- (+ SuperMApo; n = 5 mice) or vehicle-treated mouse (EAE; *n* = 4 mice) spleen or iLN cell proliferation with MOG_35-55_ peptide stimulation (15 µg/mL) assessed by BrdU incorporation. Data expressed as T cell proliferation index (MOG/medium ratio), with mean ± SEM, from a representative experiment out of 3 showing similar results; *p* values are given. **f** Proliferation of T cells isolated from EAE mice (Teff) were cultured in presence of DC pulsed with MOG_35-55_ peptide, Mycobacterium tuberculosis or CD3/CD28 antibodies, with different numbers of Tregs (Treg) isolated from the spleen of mice treated (+ SuperMApo) or not (EAE) with SuperMApo (the Treg:Teff cell ratio is indicated in the figure). Data pooled from 3 independent experiments, shown as mean ± SEM. **g** MOG-specific T cell in IL17^+^CD4^+^ (Th17), IFN-γ^+^CD4^+^ (Th1) and IFN-γ^+^CD8^+^ (Tc1) subsets from the spleen and iLN of SuperMApo- (+ SuperMApo) or vehicle-treated (EAE) mice (*n* = 4 mice per group). Data from a representative experiment out of 3 showing similar results, shown as mean ± SEM. Statistical significance was assessed by using unpaired two-tailed Student’s *t* test, with *p < 0.05, **p < 0.01, ***p < 0.001

Considering the “outside-in” paradigm of the pathophysiology of multiple sclerosis, which proposes that pathogenic T cells are activated in the periphery and then cross the BBB [35], we analyzed the spleen and inguinal lymph nodes of EAE mice 72 h after receiving or not SuperMApo and observed only marginal differences in terms of percentages (not shown) and absolute numbers of total Th1, Th17 and Treg cell subsets, as well as CD8^+^ T cells and Tc1 cell subset in both lymphoid organs (Fig. 2a, b). Nevertheless, the total number of CD4^+^ T cells was significantly increased at that time point while it goes back to normal 7 days post-treatment (not shown), which might suggest a temporary retention of CD4^+^ T cells in the spleen. We also observed neither a difference in terms of T cell apoptosis nor T cell proliferation, as attested by activated caspase-3 and Ki67 T cells expression, in lymphoid organs (Fig. 2c, d). Therefore, we investigated the autoreactive T cell response by culturing cells isolated from these lymphoid organs in the presence of the pathogenic MOG_35-55_ peptide. T cells from the spleen and inguinal lymph nodes of EAE mice treated with SuperMApo demonstrated a significant reduced proliferation in response to MOG_35-55_ peptide compared to T cells isolated from control animals (Fig. 2e). As this could result from an increase of MOG-specific Tregs in response to SuperMApo, we used MOG-specific suppressive assays. However, we were not able to detect an increase of autoantigen-specific Treg immunosuppression (Fig. 2f). When analyzing closer the MOG-specific pathogenic T cells, using MOG peptide ex vivo stimulation, we observed a reduced frequency of MOG-specific CD4^+^ and CD8^+^ T cells secreting IFN-γ (Fig. 2g), demonstrating reduced pathogenic Th1 and Tc1 cell subsets in vivo. Therefore, we conclude, that MOG-specific Th1 and Tc1 pathogenic T cell subsets are reduced in the periphery after the injection of SuperMApo pro-resolving factors.

### Pro-resolving factors administration controls EAE through induction of pro-tolerogenic macrophages

**Fig. 3:**
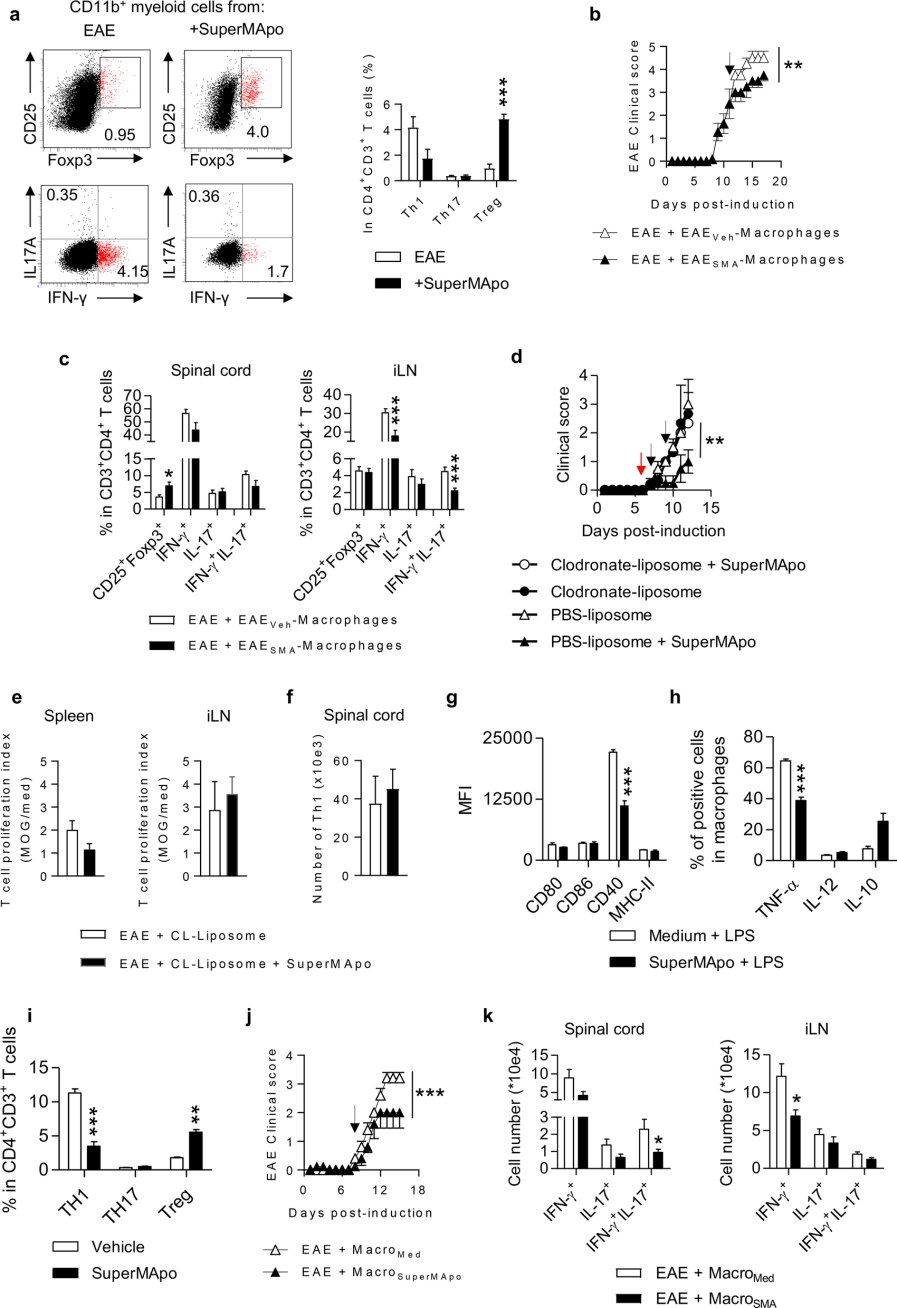
SuperMApo treatment induce pro-tolerogenic macrophages. **a** IL17^+^CD4^+^ (Th17), IFN-γ^+^CD4^+^ (Th1) and Foxp3^+^CD25^+^CD4^+^ (Treg) cell polarization by macrophages sorted from the spleen. Left panels show representative staining. Data from a representative experiment out of 3 showing similar results shown as mean ± SEM (5 mice per group). **b** Clinical score of mice treated with macrophages sorted from vehicle- (EAE + EAE_Veh_-macrophages) or SuperMApo-treated mice (EAE + EAE_SMA_-macrophages) injected at day 11 (arrow) post-induction (*n* = 4 mice). Data from a representative experiment out of 3 showing similar results, shown as mean ± SEM at each time point. **c** Percentages of T cell subsets in the spinal cord (left) and the inguinal lymph node (iLN) (right) seven days post-treatment of mice from **b** (*n* = 4 mice). Data pooled from 2 independent experiments shown as mean ± SEM. **d** Clinical score of mice treated with clodronate-loaded or PBS-loaded liposomes and then treated with vehicle or SuperMApo at day 7 and 9 (*n* = 3–4 mice per group). Data from a representative experiment out of 2 showing similar results, shown as mean ± SEM. The red arrow marks the injection of liposomes while the black arrows mark the injection of SuperMApo. **e** T cell proliferation index after MOG_35-55_ peptide stimulation, from mice from **d**. **f** Th1 cell numbers of mice from **d**. Expression of co-stimulatory molecules **g** and intracellular pro-inflammatory cytokines **h** after LPS stimulation on macrophages during 24 h. Data from a representative experiment out of 3 showing similar results, shown as mean ± SEM. **i** Frequencies of Th1, Th17 and Treg cells after co-culture of naïve CD4^+^ T cells with macrophages pretreated with SuperMApo or vehicle during 24 h. Data from a representative experiment out of 2 showing similar results, shown as mean ± SEM. **j** Clinical score of EAE mice receiving at day 8 post-EAE induction (arrow) macrophages pretreated by SuperMApo (EAE + Macro_Med_) or medium (EAE + Macro_SuperMApo_) for 24 h. Data from a representative experiment out of 2 showing similar results, shown as mean ± SEM. **k** Numbers of CD4^+^ T cell subsets in the spinal cord (left) or iLN (right) from mice from **j**, 7 days after macrophage injection. * = *p* < 0.05, ** = *p* < 0.01, *** = *p* < 0.001, unpaired two-tailed Student’s *t* test (**a**, **c**, **e**–**i**, **k**) or two-way ANOVA with Bonferroni posttest (**b**, **d**, **j**)

As our data indicate that CNS infiltrating macrophages were reduced and less inflammatory after SuperMApo treatment (Fig. 1d–g) and as macrophages reportedly play key roles in controlling EAE, notably by modulating the Th1-Th17/Treg balance [36–38], and are key players in the resolution phase of inflammation due to their microenvironment and tissue location plasticity [21], we further investigated the impact of SuperMApo on their behavior. Even though no Treg increase was observed in SuperMApo-treated EAE mice (Fig. 1d) nor *ex vivo* MOG-specific Treg activity (Fig. 2f), spleen macrophages isolated 72 h after SuperMApo treatment demonstrated the capacity to favor Treg rather than Th1 cell polarization of naive CD4^+^ T cells *ex vivo* (Fig. 3a). That pro-tolerogenic profile of macrophages acquired *in vivo* through SuperMApo treatment was further confirmed by the adoptive transfer of macrophages isolated from the spleen of SuperMApo-treated EAE mice into EAE mice with moderate disease activity, in which they significantly modulated the severity of the disease compared to macrophages collected from vehicle-treated EAE mice (Fig. 3b and Additional file 1: Fig. S5a). The disease modulation was associated with a significantly higher Treg percentage in the spinal cord and a reduced rate of IFN-γ^+^ and IFN-γ^+^/IL-17^+^-producing CD4^+^ T cell percentages in the iLN (Fig. 3c). To confirm a central role of macrophages in SuperMApo-mediated EAE control, we used clodronate-loaded liposome injection, as previously described [29] to deplete them prior to treatment. Macrophage depletion significantly inhibited the therapeutic efficacy of SuperMApo treatment compared to control EAE (Fig. 3d and Additional file 1: Fig. S5b). Consistently with this, T cells from the spleen and the iLN of mice depleted of macrophages and treated with SuperMApo did not show a reduced proliferation to MOG_35-55_ peptide stimulation (Fig. 3e), nor a reduction of spinal cord Th1 cell numbers (Fig. 3f).

To confirm that SuperMApo was able to modulate macrophage functions, we performed a series of *in vitro* experiments. Macrophages treated *in vitro* with SuperMApo first showed resistance to LPS-induced maturation as attested by lower expression of CD40 and intracellular TNF-α content (Fig. 3g, h). Second, they favored Treg induction rather than Th1 cell polarization when cultured with naive CD4^+^ T cells (Fig. 3i). Last, and most importantly, when adoptively transferred into EAE mice, they controlled the disease (Fig. 3j) by reducing the generation of pathogenic IFN-γ^+^ producing cells in inguinal lymph nodes and IFN-γ^+^/IL-17^+^ CD4^+^ T cells in the spinal cord (Fig. 3k). Collectively, these data demonstrate that SuperMApo modulates macrophages toward a pro-tolerogenic profile, critical to control EAE disease.

### Pro-resolving factors-induced macrophages limit CXCR3-dependent Th1 cell migration

**Fig. 4.**
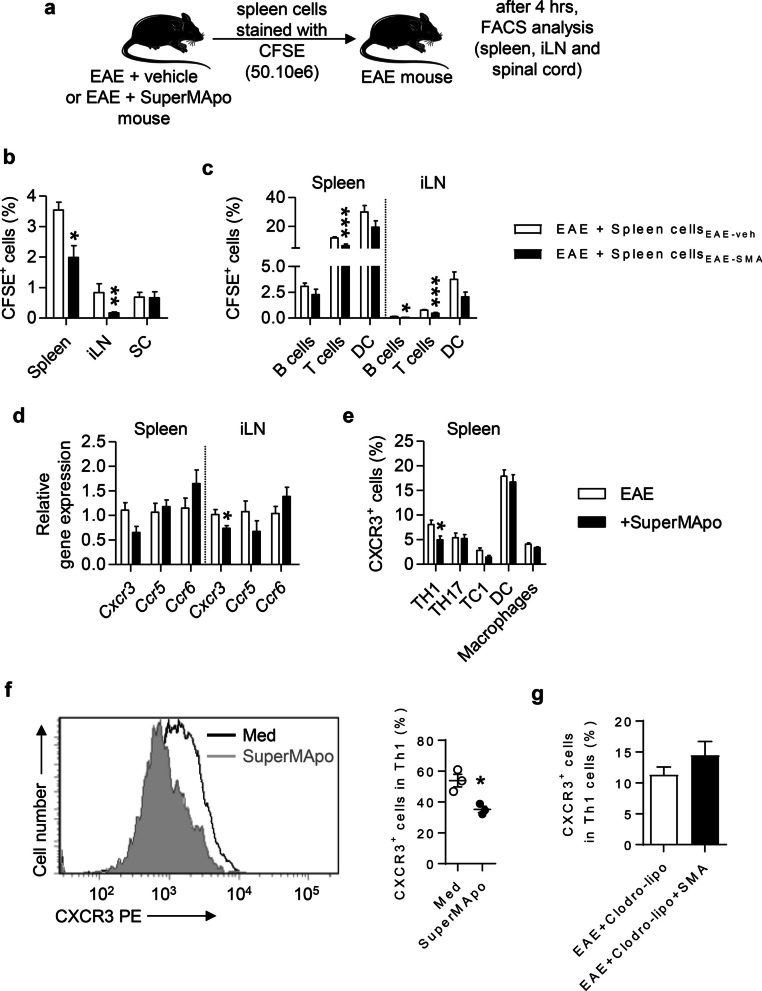
Macrophages primed by resolution type secretome exposure modulate the expression of CXCR3 on Th1 cells. **a** Experimental scheme of the transfer of CFSE-labeled spleen cells from EAE mice treated or not by SuperMApo. **b** Percentages of CFSE positive cells from different origin (EAE + vehicle or EAE + SuperMApo mice) in the spleen, inguinal lymph node (iLN) and spinal cord (SC). Data pooled from 2 independent experiments. **c** Percentage of CFSE positive B, T and dendritic cells (DC) in the spleen and iLN of mice as in **b**. Data pooled from 2 independent experiments shown as mean ± SEM. **d** Expression of *Cxcr3*, *Ccr5* and *Ccr6* mRNA in the spleen and iLN cells issued from vehicle- (EAE) or SuperMApo-treated mice (+ SuperMApo). Data from a representative experiment out of 3 showing similar results, shown as mean ± SEM, 4 mice per group. **e** Expression of CXCR3 on Th1, Th17, Tc1, DC and macrophages in the spleen of mice as in **d**. Data from a representative experiment out of 3 showing similar results, shown as mean ± SEM, 5 mice per group. **f** Expression of CXCR3 in Th1 cells after co-culture of naive CD25^‒^CD4^+^ T cells with macrophages pretreated by SuperMApo or control medium, depicted as representative histograms (left) or cumulated including mean ± SEM (right). Data from a representative experiment out of 3 showing similar results. **g** Expression of CXCR3 on spleen Th1 cells from EAE mice depleted for phagocytes (EAE + Clodro-lipo) treated or not with SuperMApo (+ SMA). Data pooled from two independent experiments shown as mean ± SEM from 4 to 5 mice per group. * = *p* < 0.05, ** = *p* < 0.01, *** = *p* < 0.001, unpaired two-tailed Student’s t test

Since leukocyte migration into the CNS is one of the major pathogenic feature of EAE development [39, 40], we next tested whether SuperMApo-reprogrammed macrophages may modulate the peripheral immune response during ongoing EAE by limiting T cell migration. When analyzing the migratory capacities of spleen cells from SuperMApo-treated EAE mice transferred to EAE mice, we observed a decreased ability of the cells to migrate to the secondary lymphoid organs (Fig. 4a, b), and this mostly concerned T cells (Fig. 4a–c). When analyzing chemokine mRNA expression in the spleen and inguinal lymph nodes of SuperMApo-treated EAE mice, we detected a reduced expression of *Cxcr3* mRNA, but not of *Ccr5* and *Ccr6* mRNA, compared to control animals (Fig. 4d) which was restricted to Th1 cells as confirmed at the protein level (Fig. 4e). We indeed confirmed *in vitro* that SuperMApo-treated spleen macrophages induced less CXCR3 expression on Th1 cells (Fig. 4f). Furthermore, the percentage CXCR3^+^ Th1 cells in mice depleted for phagocytes was not reduced despite SuperMApo treatment (Fig. 4g). Altogether, these data demonstrate that SuperMApo treatment decreases the ability of Th1 cells to migrate by reducing their expression of CXCR3 through macrophage reprogramming.

### Pro-resolving factors reprogram macrophages among CD11b^+^ cells via modulation of DNA methylation

**Fig. 5:**
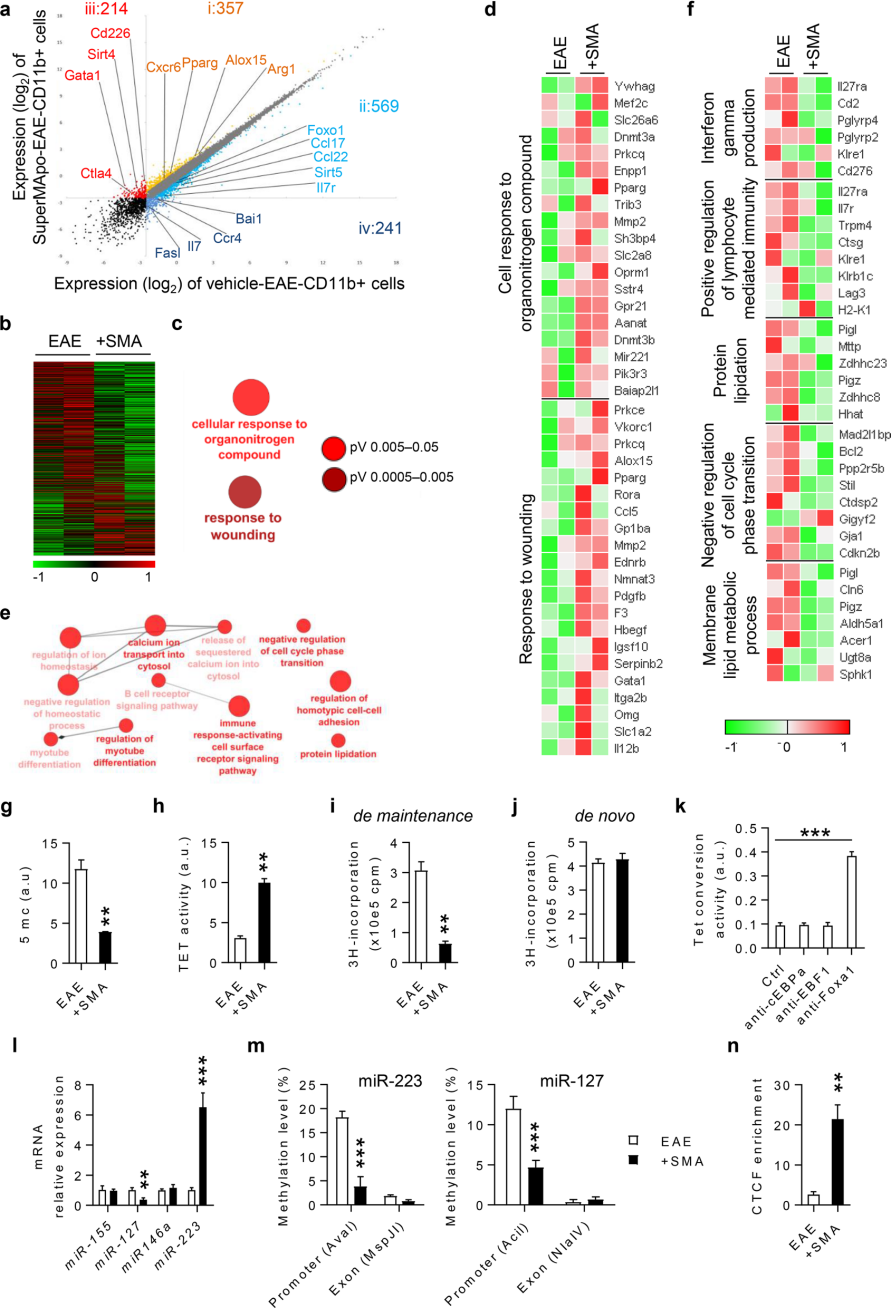
SuperMApo treatment reprograms macrophages among CD11b^+^ myeloid cells toward a pro-tolerogenic profile at the epigenetic level. **a** Gene expression (log2 FPKM transformed) ordered based on their fold change: i, in orange 357 genes with a change in expression of 1.5-fold or more, (ii, in light blue, 569 genes with a fold change of 0.67 or below, iiii, in red, 214 genes expressed only in CD11b^+^ cells from SuperMApo-treated mice and iv, in dark blue, 241 genes expressed only in CD11b^+^ cells from vehicle-treated mice. **b** Expression (log2 FPKM transformed, normalized, mean centered) of all 12,049 genes in mouse CD11b^+^ cells, clustered from lowest to highest fold change. **c** Network analysis of gene-ontology pathway enrichment on all 357 genes with a fold change of 1.5 or more and all 214 genes only expressed in CD11b^+^ cells from SuperMApo-treated mice with a *P* < 0.05. **d** Expression (log2 FPKM transformed, normalized, mean centered) of genes corresponding to selected pathways found in the network analysis enrichment on all 357 genes with a fold change of 1.5 or more and all 214 genes only expressed in CD11b^+^ cells from SuperMApo-treated mice. **e** Network analysis of gene-ontology pathway enrichment on all 569 genes with a fold change of 0.67 or below and all 241 genes only expressed in CD11b^+^ cells from vehicle-treated mice. **f** Expression (log2 FPKM transformed, normalized, mean centered) of selected genes corresponding to selected pathways found in the network analysis enrichment on all 569 genes with a fold change of 0.67 or below and all 241 genes only expressed in CD11b^+^ cells from vehicle-treated mice. Data obtained from 2 independent experiments from a pool of 5 mice per condition. **g** Total level of 5-methylcytosine observed in CD11b^+^ cells sorted from mice 72 h post-treatment. TET activity (**h**), *de maintenance* (**i)** and de novo (**j)** methyltransferase activity in CD11b^+^ cells from **g**. **k** TET conversion activity in respective CD11b^+^ cells. **l** Expression of miRNA-155, -127, -146a and -223 in CD11b^+^ cells from **g**. **m** Levels of methylation of genes coding for miR-223 (left) and miR-127 (right) in CD11b^+^ cells from **g**. **n** CTCF enrichment on region 1 of miR-127 promoter from **g**. Data pooled from three independent experiments expressed as mean ± sem, 5 mice per group. ** = *p* < 0.01, *** = *p* < 0.001, unpaired two-tailed Student’s *t* test

To further confirm macrophage reprogramming, a transcriptome analysis was performed on CD11b^+^ cells sorted from the spleen of SuperMApo- and vehicle-treated EAE mice, at 72 h post-treatment (Additional file 1: Fig. S6). As shown in Fig. 5, among the 571 up-regulated genes in SuperMApo-EAE-CD11b^+^ cells (*vs* vehicle-EAE-CD11b^+^ cells), we found the M2-specific markers *Arg1*, *Pparg*, *Cd226* and *Alox15* expressed by pro-resolving macrophages (Fig. 5a, b). Additionally, among the 810 down-regulated genes in SuperMApo-EAE-CD11b^+^ cells, we found the pro-inflammatory genes *Fasl*, *Il7*, *Ccr4* and *Ccl22* (Fig. 5a, b). Signaling pathway analysis demonstrated the upregulation of pathways associated with wound healing (a major feature of inflammation resolution) in SuperMApo-EAE-CD11b^+^ cells (Fig. 5c, d). Pathways involved in cellular function (i.e., protein lipidation, regulation of cell cycle phase transition, membrane lipid metabolic process) and immune activation were found to be down-regulated in SuperMApo-EAE-CD11b^+^ cells (Fig. 5e, f). Interestingly, we observed that macrophages isolated from SuperMApo-treated EAE mice also demonstrated a decreased phosphorylation of IKK-β, IκB and p65 subunit, and a decreased percentage of p65 subunit in the nucleus (Additional file 1: Fig. S7a). These results suggest that SuperMApo induces macrophage reprogramming at the transcriptomic level, inducing pro-resolving properties to control ongoing EAE.

Interestingly, at the DNA level, we found that spleen CD11b^+^ cells from SuperMApo-treated EAE mice showed a decreased global level of 5-methylcytosine (Fig. 5g) which was associated with an increase in TET enzyme activity, and particularly of the *de maintenance* methylation activity (but not *de novo* methylation) (Fig. 5h–j). Additional experiments performed with antibodies directed against TET1, TET2 and TET3 suggested that TET1 is responsible for the increase of global TET activity since only TET1 antibody limited the TET activity (Additional file 1: Fig. S7b). When using an anti-Foxa1 antibody, the TET conversion activity was found modulated, but not with antibodies targeting c-EBPa or EBF1, demonstrating that the Foxa1–TET complex was responsible for CD11b^+^ cells DNA decreased methylation (Fig. 5k), and notably of the FOXA1-targeted gene family. DNA methylation and miRNA expression mutually regulate each other which has dramatic consequences in the control of inflammation [41, 42]. Notably, it has been reported that two miRNAs are particularly important in regulating macrophage responses. These being miR-223, an anti-inflammatory miRNA [43, 44], and miR-127 a pro-inflammatory miRNA [45]. Consequently to these DNA methylation modifications induced by SuperMApo in CD11b^+^ cells, the anti-inflammatory *miR-223* expression was found to be increased, and associated to the demethylation of *miR-223* promoter (Fig. 5l, m). In addition, the pro-inflammatory *miR-127* expression was decreased despite the demethylation of its promoter (Fig. 5l, m), but explained by its proximity to the CCCTC-binding factor (CTCF) transcriptional repressor binding site, which was observed enriched by ChIP assay in SuperMApo-treated CD11b^+^ cells (Fig. 5n). Thus, macrophage reprogramming induced by SuperMApo treatment during ongoing EAE occurs through the control of DNA methylation, targeting FOXA1-targeted gene family, as well as the repression of the pro-inflammatory *miR-127* and the increase of the anti-inflammatory *miR-223*.

These data support a durable reprogramming of macrophages by SuperMApo treatment within a disease environment, which is sustained even after adoptive transfer.

## Discussion

Resolution of inflammation is impaired in numerous chronic inflammatory diseases, including MS [46] and engaging the resolution of inflammation could be a promising approach to develop new treatment modalities [11, 13]. Here, we demonstrated that the injection of pro-resolutive factors released by macrophages after efferocytosis allows the control of EAE progression by targeting macrophages through epigenetic reprogramming to obtain durable pro-tolerogenic properties.

Several conclusions can be drawn from our study. The first relates to the efferocytosis factors released by macrophages eliminating apoptotic cells, which seem key for inducing peripheral macrophage reprogramming toward a pro-resolving profile, and confirm the critical role of efferocytosis in the maintenance [22, 23] and induction of tolerance [29, 47]. In normal tissue environments where inflammation resolution is executed, efferocytosis reprograms macrophages to produce pro-resolving and healing factors to initiate the return to homeostasis [22, 23]. Our data suggest further that efferocytic macrophages are communicating not only with neighboring but also with peripheral macrophages through the emitted factors to the extent of reprogramming them durably and at the epigenetic level, promoting a pro-resolution phenotype. These factors are also able to propagate macrophage reprogramming in vivo even in an inflammatory environment. No modulation of the course of EAE, nor the induction of macrophage reprogramming was observed when factors released by apoptotic cells alone or non-efferocytic macrophages, or their combination were used. This demonstrates that factors specifically released by efferocytic macrophages, or factors increasing significantly after efferocytosis, are implicated in macrophage reprogramming. For instance, TGF-β is released by apoptotic cells themselves [48] and TGF-β production by macrophages is markedly increased after efferocytosis [19, 29]. As of yet, we were not able to identify the individual factors responsible for macrophage reprogramming, but previous work demonstrated that factors released after efferocytosis are numerous and act in synergy. In our experience, neither the use of the six anti-inflammatory factors CXCL2, CCL5, CCL22, IL-1RA, IL-10 and TGF-β found in SuperMApo (at their natural concentration or enriched threefold), nor the use of the 5 factors found to be complexed with TGF-β in SuperMApo were able to induce macrophage reprogramming or reproduce the therapeutic effect of SuperMApo in ongoing arthritis [26]. Similarly, the use of TGF-β, IGF-I and VEGF present in SuperMApo and participating in colon mucosal healing in the experimental model of colitis failed to induce wound healing in ongoing colitis [27]. Therefore, the specific combination of molecules responsible for the propagation of macrophage reprogramming and participating in epigenetic modifications of these cells remains to be identified.

Second, reduced inflammation and demyelination in the spinal cord seemed to be due to the modulation of peripheral immune responses rather than induced directly within the CNS. This includes a decrease of inflammatory T cell migration to the CNS via the modulation of CXCR3 expression on Th1 cells and the reduction of pathogenic Th17 and Th1 responses in the spleen and iLN. SuperMApo did not favor a remodeling of the BBB, since we did not observe a decrease of BBB permeability to EB dye after SuperMApo treatment nor an increase of *occludin*, *claudin-5*, *tight junction protein 1* and *F11 receptor* mRNA levels. This could be attributed to the experimental model of EAE used that includes Pertussis toxin injection to alter the BBB but, in a demyelination model using cuprizone injection [49], we could not detect accelerated remyelination by SuperMApo treatment neither (data not shown). This contrasts with the tissue repair properties of SuperMApo observed in experimental arthritis [26] or colitis [27]. However, the regulatory mechanisms of SuperMApo might not only be disease-specific [26–28] but could also be site-specific (i.e., inflamed joints, intestine or CNS) with the targeting of different T cell, myeloid cells or DC subsets [26–28] (our current experiences). We were surprised to not be able to detect a modulation of microglial cell activation (CNS-resident macrophages [50]), a common feature of MS and EAE [1], after SuperMApo treatment. A deeper evaluation of the response of microglia to SuperMApo is subject of further investigations. In contrast to microglia, CNS infiltrating macrophages showed pro-tolerogenic properties, confirming the effect of SuperMApo in the periphery. This hypothesis is also supported by the depletion of peripheral macrophages using i.v. clodronate-loaded liposome infusion that prevented the effect of SuperMApo, as well as by the adoptive transfer of splenic macrophages, which recapitulated the effects of SuperMApo. It has to be noted here that one of the limitations of our study is that the MOG peptide model of EAE is more reflective of acute neuroinflammation and that our work will need to be confirmed in other models reflecting more closely chronic neuroinflammation like the PLP model of EAE into SJL/J mice.

Third, splenic macrophages are mandatory in the control of ongoing CNS inflammation induced by pro-resolving factors in EAE mice. This is supported by the depletion of phagocytes using i.v. clodronate-loaded liposome injection that totally abrogated the therapeutic effect of SuperMApo treatment. Our data suggest that macrophages interact with T cells after SuperMApo treatment, since macrophage depletion restores T cell proliferation to the auto-antigen MOG_35-55_. Adoptive transfer of splenic CD11b^+^ cells isolated from EAE mice treated with SuperMApo confirms the critical role of these cells with the reduction of EAE clinical score and of iLN Th1 cells. Furthermore, this transfer induced an increase of Treg in the spinal cord. Concerning the role of Treg after SuperMApo treatment, while we were not able to detect a MOG_35-55_-specific Treg increase nor higher Treg suppressive activity, the depletion of Treg in Foxp3-DTR EAE mice before SuperMApo treatment abrogated the control of EAE by SuperMApo treatment (data not shown). Moreover, SuperMApo-reprogrammed macrophages induced ex vivo Treg generation. Altogether, this suggests that tolerogenic macrophages may favor the emergence of Treg that are necessary to mediate long-term tolerance, but at a level that is not detectable by our assays. Alternatively, Treg numbers isolated from the CNS or the lymph nodes are too low to perform suppression assays. SuperMApo-reprogrammed macrophages do not affect only Treg, but also pathogenic Th1 and Th17 cells. In particular, these macrophages decrease CXCR3 expression on Th1 cells and T cell migration into the CNS, confirming the importance of this chemokine receptor in T cell migration into the CNS during EAE [40].

Fourth, macrophages are highly plastic cells extremely sensitive to their microenvironment [51]. A vast “spectrum” of functions characterized by an array of macrophage phenotypes/subtypes can be exerted by these innate immune cells [52]. In the current study, spleen macrophages exposed to pro-resolving factors in an inflammatory setting (*i.e*., EAE or LPS) showed a decreased activation state both in vitro and in vivo. This is attested by a down-regulation of the NF-κB pathway. Our transcriptomic data confirm the pro-resolving phenotype of CD11b^+^ cells, with a down-regulation of signaling pathways involved in immune activation. The pathway “response to wounding” is also found up-regulated, as well as numerous markers that are associated with the “alternatively activated”/M2 phenotypes (e.g., *Arg1*, *Alox15*, and *Pparg*). Arginase-1 is a well-described pro-resolving/M2 macrophage marker—at least in mice [53]. Engagement of the IL-4 receptor-α signaling pathway together with the recognition by mouse macrophages of apoptotic cells via AXL and MERTK receptor tyrosine kinases induces *Alox15* transcripts [54]. The arachidonate 15-lipoxygenase (ALOX15) is also a marker of human M2 macrophages [55]. PPAR-γ activation in macrophages is essential for the expression of genes controlling the M2 responses [56], and miR-223 has been shown to be a critical mediator of PPAR-γ-induced M2 macrophage polarization [57]. In line with the epigenetic regulation of pro-resolving CD11b^+^ cells, an increase of *Dnmt3a* and *Dnmt3b* transcripts in SuperMApo-reprogrammed CD11b^+^ cells was identified in our transcriptomic data. DNA methyltransferase 3A (DNMT3A), has been recently shown to play a crucial role in the promotion of tissue resolution by efferocytic macrophages [20]. The M1 marker CD80 together with MHC class II molecules are decreased in CNS macrophages of EAE mice treated with SuperMApo, confirming this skewed pro-resolving profile. This specific control by SuperMApo occurs through epigenetic modifications, suggesting a stable phenotype. Indeed, *miR-223* expression is tightly controlled by DNA methylation through an increase of TET protein activity in a Foxa1-dependent axis. This lead to a skewed pro-resolving profile, since we found an upregulation of *miR-223* expression that is compatible with a *Pparg* gene upregulation detected by transcriptomic analysis and with a pro-resolving phenotype (see above [56, 57]). It has to be noted that the transcriptomic analysis was performed on CD11b^+^ cells and with that may contain different macrophage subtypes as well as other CD11b^+^ cells.

Altogether, our data demonstrate that the factors released by macrophages after efferocytosis participate in a deep macrophage reprogramming locally (*i.e*., CNS), and in the periphery. Which, in turn, shapes adaptive and innate cell responses to induce and maintain tolerance.

## Conclusion

To conclude, our data demonstrate that injection of pro-resolving factors released by efferocytic macrophages are able to therapeutically modulate immune responses in ongoing EAE through a specific and prolonged control of macrophage functions via their epigenetic reprogramming.

Our preclinical data presented here, will need further validation before being moved to the clinic, but show encouraging results of the potential of secretome-based therapies targeting epigenetic reprogramming, in line with applied resolution pharmacology [58]. Secretome-based drugs represent a new therapeutic modality [59] and have gained momentum in recent years, as they are preserving the potential of the paracrine action of cellular therapy, while overcoming the survival or erroneous differentiation complications of transplanted cells in the host tissue. The clinical investigations of cell secretomes have already started and are supported by regulatory agencies, evidenced by the multiple clinical trials currently registered at global clinical trial registries (https://www.clinicaltrialsregister.eu/ and clinicaltrial.gov accessed April 2023). Our data have shown that the therapeutic yet epigenetic reprogramming of myeloid cells is possible in the context of neuroinflammation where multiple pathways are dysregulated. This approach, once proven safe and effective in the upcoming clinical evaluation, could overcome not only the limitations associated with current therapeutics but open an entirely new chapter in medical care.

### Supplementary Information

**Additional file 1: Fig. S1.** Apoptotic cell supernatant and macrophage supernatant injections do not improve EAE. **Fig. S2.** Gating strategy. **Fig. S3.** SuperMApo treatment modulates CNS infiltrating macrophage but not microglia activation profiles. **Fig. S4.** SuperMApo treatment does not modulate directly the blood brain barrier. **Fig. S5.** Macrophage detection from tissues. **Fig. S6.** FACS analysis of CD11b spleen cell sorting from EAE mice receiving or not SuperMApo treatment. **Fig. S7.** SuperMApo treatment decreases macrophage inflammatory state by blocking NF-κB activation.

## Data Availability

All data generated or analyzed during this study are included in this published article and its supplementary information file.

## Abbreviations

CNS: Central nervous system
EAE: Experimental autoimmune encephalomyelitis
MS: Multiple sclerosis
SPM: Specialized pro-resolving lipid mediators
DEL-1: Developmental endothelial locus-1
Treg: Regulatory T cells
DSS: Dextran sodium sulfate
APC: Antigen-presenting cells
MOG_35-55_: Myelin oligodendrocyte glycoprotein peptide 35–55
BBB: Brain blood barrier
DC: Dendritic cells
