# Supplementary material for: Amelioration of experimental autoimmune encephalomyelitis by in vivo reprogramming of macrophages using pro-resolving factors
Source: J Neuroinflammation. 2023 Dec 20;20:307. doi: 10.1186/s12974-023-02994-5 (PMC10734130; doi:10.1186/s12974-023-02994-5)
Supplement: Supplementary file 1 — Additional file 1: Fig. S1. Apoptotic cell supernatant and macrophage supernatant injections do not improve EAE. Fig. S2. Gating strategy. Fig. S3. SuperMApo treatment modulates CNS infiltrating macrophage but not microglia activation profiles. Fig. S4. SuperMApo treatment does not modulate directly the blood brain barrier. Fig. S5. Macrophage detection from tissues. Fig. S6. FACS analysis of CD11b spleen cell sorting from EAE mice receiving or not SuperMApo treatment. Fig. S7. SuperMApo treatment decreases macrophage inflammatory state by blocking NF-κB activation. [file 12974_2023_2994_MOESM1_ESM.pdf]

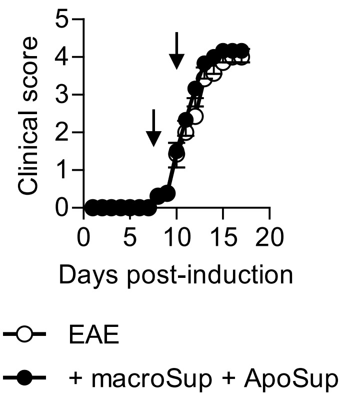

FIGURE S1. Apoptotic cell supernatant and macrophage supernatant injections do not improve EAE. Clinical score of EAE mice treated or not (EAE) twice (black arrows) with supernatant of apoptotic cells plus the supernatant of macrophages (1 mL) (+ macroSup + ApoSup). Data from a representative experiment out of 2 showing similar results, shown as mean +/- SEM, with 5 mice per group. Statistical significance was assessed using ANOVA two-way and Bonferroni posttest

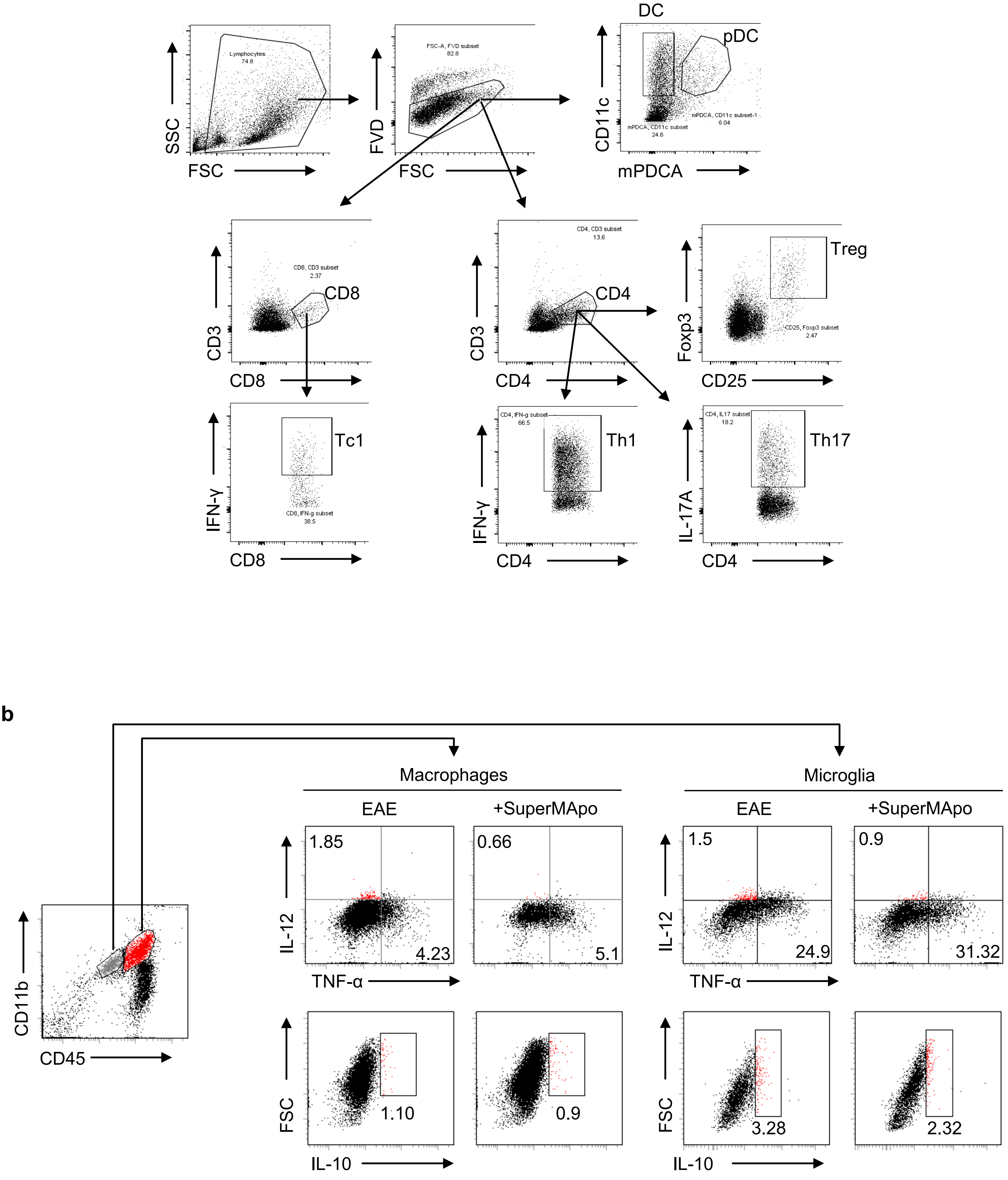

FIGURE S2. Gating strategy. (**a**) Gating strategy for immune cell analysis within the CNS. (**b**) Gating strategy for CNS macrophages and microglia analysis, gated from FSC/SSC and FSC/FVD dotplots.

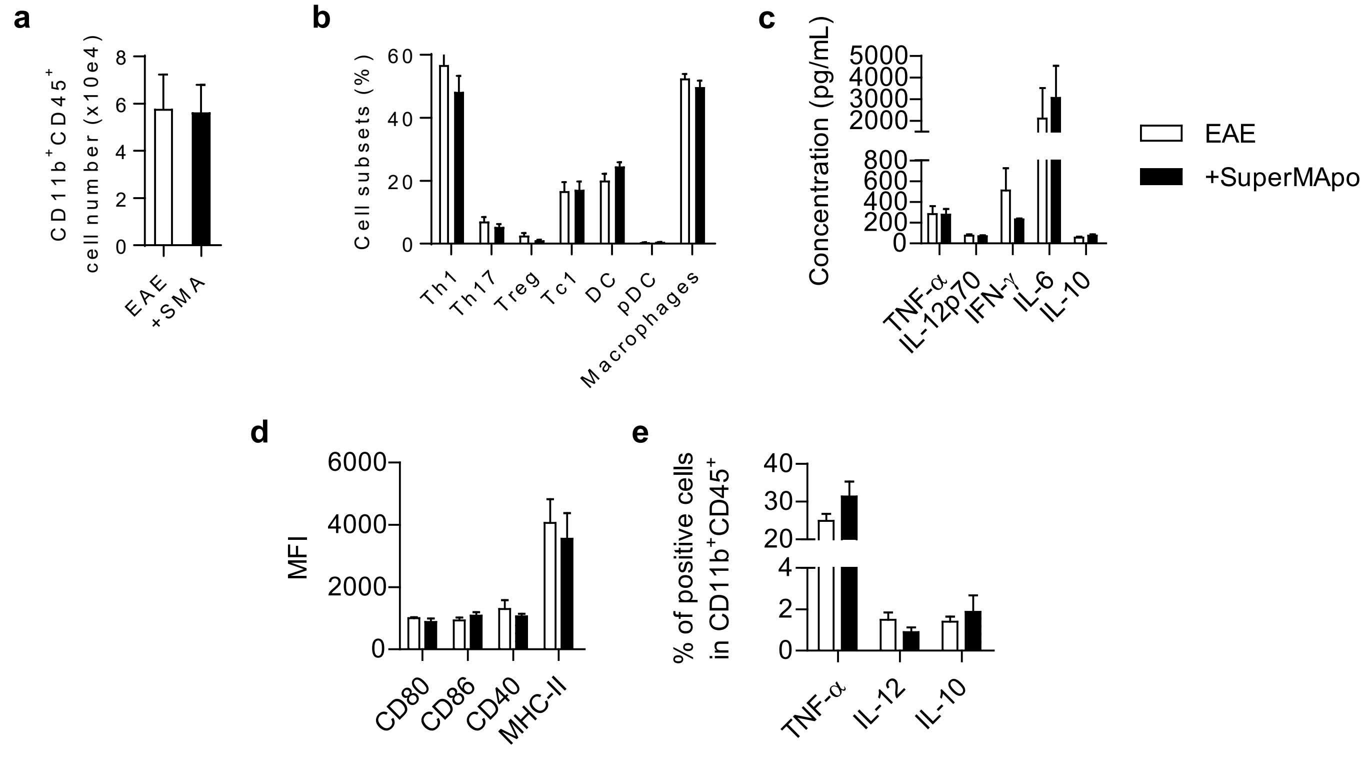

FIGURE S3. SuperMApo treatment modulates CNS infiltrating macrophage but not microglia activation profiles. (**a**) Absolute number of $CD11b^{med}CD45^{+}$ microglia in spinal cords of EAE mice 72 h after SuperMApo (+SAM) or vehicle (EAE) injection. Data representative of 3 independent experiments shown as mean +/- SEM with 5 mice per group. (**b**) Percentages of CNS cell subsets in mice from **a**. (**c**) Spinal cord fluid concentrations of TNF-α, IL-12p70, IFN-γ, IL-6 and IL-10 in mice from **a**. (**d**) Mean Fluorescence Intensity (MFI) of CD80, CD86, CD40 and MHC-II expressed by microglia in mice from **a**. (**e**) Percentages of TNF-α, IL-12 and IL-10 positive cells microglia in the spinal cords of mice from **a**. Student's t Test.

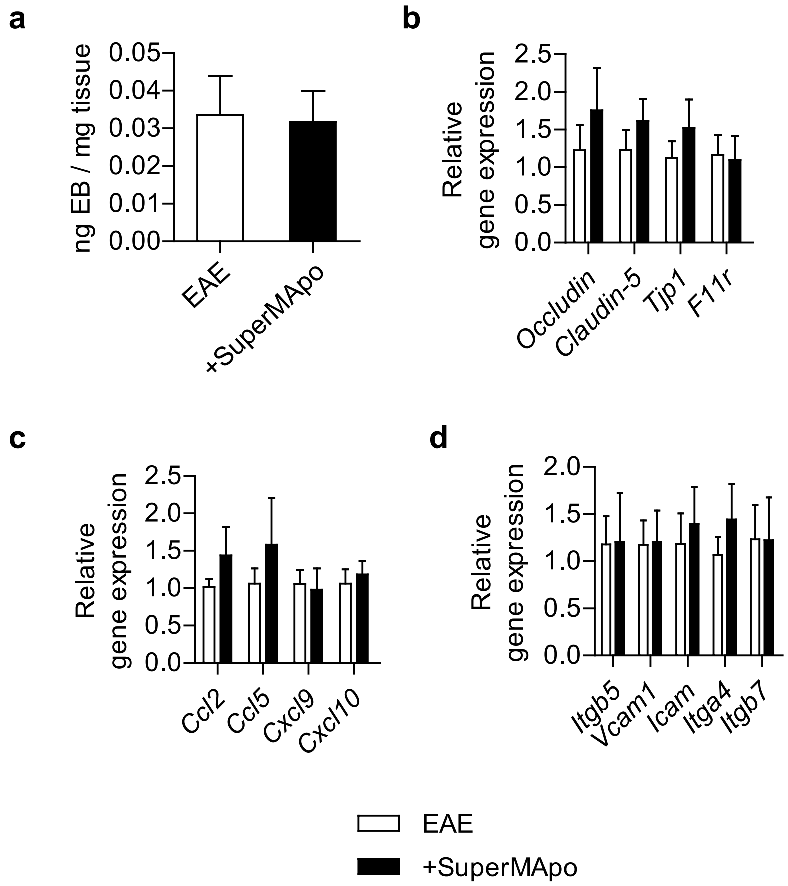

FIGURE S4. SuperMApo treatment does not modulate directly the blood brain barrier. (**a**) Evan's Blue (EB) dye quantification in EAE mice receiving SuperMApo or not (EAE) (n= 5 mice). Intravenous Evans Blue (EB) dye injections were done 72 hours post SuperMApo or control treatment at an approximate EAE score of 2. Data are given as ng of EB/mg of tissue, 5 mice per group. Data representative of 3 independent experiments shown as mean +/- SEM. Spinal cord of EAE mice receiving SuperMApo or not (EAE) were analysed for the mRNA expression of *Occludin*, *Claudin5*, *Tjp1*and *F11r* (**b**), *Ccl2*, *Ccl5*, *Cxcl9* and *Cxcl10* (**c**) and *Itgb5*, *Itgb7*, *Vla-4*, *Icam-1* and *Vcam* (**d**). Data are representative of 3 independent experiments, 5 mice per group, and shown as mean +/- SEM. Student's t Test.

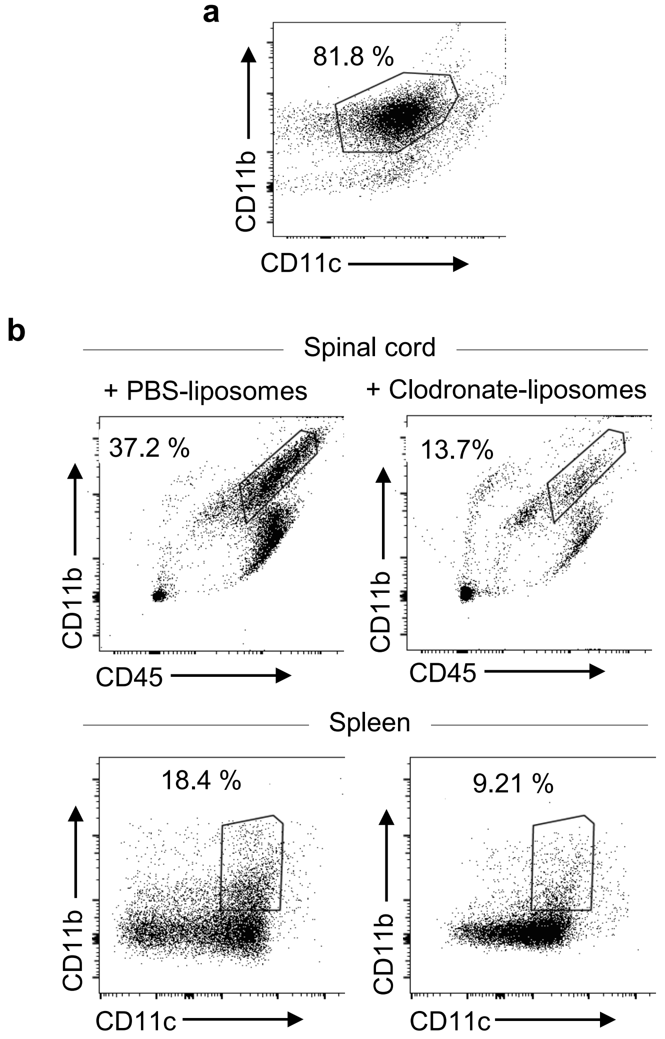

FIGURE S5. Macrophage detection from tissues. (**a**) Purity of $CD11b^+$ myeloid cells sorted from the spleens for adoptive transfer experiments. (**b**) $CD11b^+$ myeloid cells percentage from the spinal cord and spleen of EAE mice 24 hours after receiving clodronate- or PBS-loaded liposomes

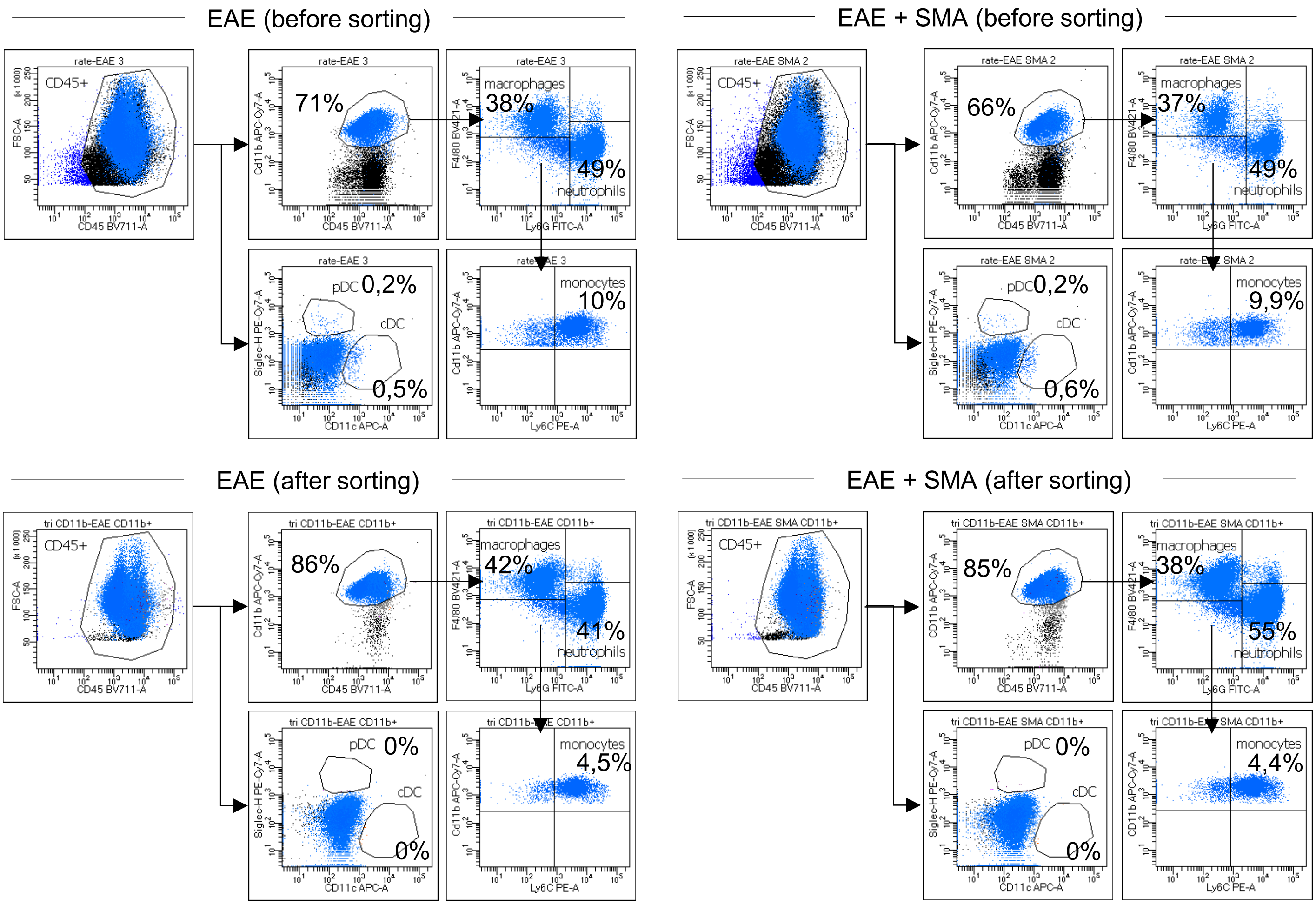

Figure S6. FACS analysis of CD11b spleen cell sorting from EAE mice receiving or not SuperMApo treatment. EAE mice were sacrificed 72h after SuperMApo (SMA) treatment and spleen cells were collected and stained before and after CD11b cell magnetic isolation. $CD11b^{+}CD45^{+}$ cells percentage corresponds to total cells; other percentages correspond to $CD11b^{+}CD45^{+}$ cells.

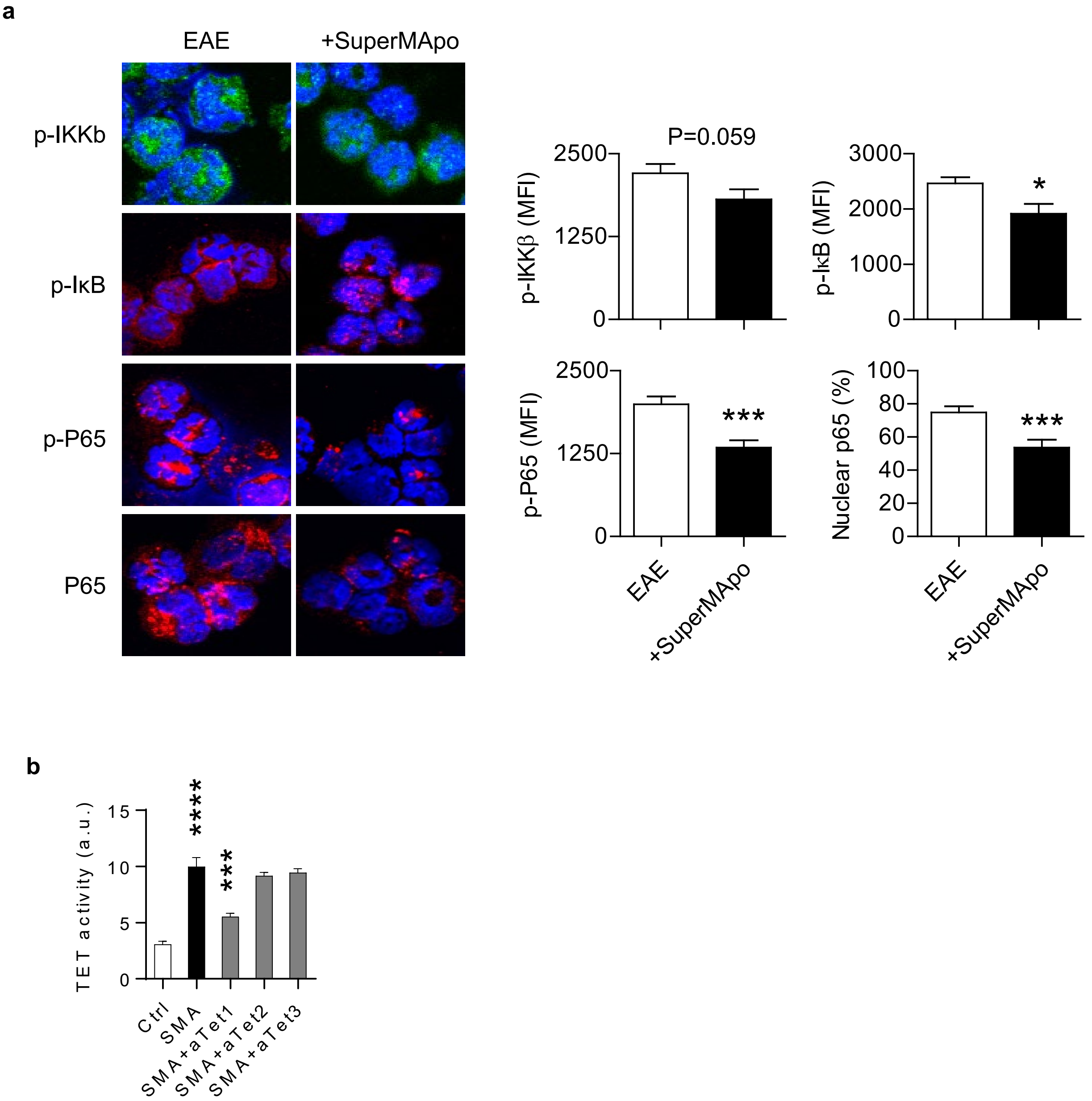

FIGURE S7. SuperMApo treatment decreases macrophage inflammatory state by blocking NF-κB activation. (**a**) Immunofluorescence staining of p-IKKb, p-IκB, p-P65 and P65 subunits on $CD11b^+$ myeloid cells sorted from the spleen of SuperMApo- or vehicle-treated EAE mice. Data, representative of three independent experiments, are shown as representative pictures (left panels) or cumulative bar graphs (right panels) as mean +/- SEM. *=p<0.05, ***=p<0.001, unpaired two-tailed Student's t test. (**b**) TET activity in $CD11b^+$ myeloid cells from EAE mice receiving vehicle (Ctrl) or SuperMApo (SMA) in the presence or not of blocking antibodies against TET1 (aTet1), TET2 (aTet2) or TET3 (aTet3). Data are shown as mean +/- SEM. ***=p<0.001, ****=p<0.0001, one-way ANOVA plus Tukey's multiple comparisons test.
